# Microsatellites Reveal a High Population Structure in *Triatoma infestans* from Chuquisaca, Bolivia

**DOI:** 10.1371/journal.pntd.0000202

**Published:** 2008-03-26

**Authors:** Juan Carlos Pizarro, Lauren M. Gilligan, Lori Stevens

**Affiliations:** 1 Facultad de Bioquímica, Universidad de San Francisco Xavier de Chuquisaca, Sucre, Bolivia; 2 Department of Biology, University of Vermont, Burlington, Vermont, United States of America; René Rachou Research Center, Brazil

## Abstract

**Background:**

For Chagas disease, the most serious infectious disease in the Americas, effective disease control depends on elimination of vectors through spraying with insecticides. Molecular genetic research can help vector control programs by identifying and characterizing vector populations and then developing effective intervention strategies.

**Methods and Findings:**

The population genetic structure of *Triatoma infestans* (Hemiptera: Reduviidae), the main vector of Chagas disease in Bolivia, was investigated using a hierarchical sampling strategy. A total of 230 adults and nymphs from 23 localities throughout the department of Chuquisaca in Southern Bolivia were analyzed at ten microsatellite loci. Population structure, estimated using analysis of molecular variance (AMOVA) to estimate F_ST_ (infinite alleles model) and R_ST_ (stepwise mutation model), was significant between western and eastern regions within Chuquisaca and between insects collected in domestic and peri-domestic habitats. Genetic differentiation at three different hierarchical geographic levels was significant, even in the case of adjacent households within a single locality (*R*_ST_ = 0.14, *F*_ST_ = 0.07). On the largest geographic scale, among five communities up to 100 km apart, *R*_ST_ = 0.12 and *F*_ST_ = 0.06. Cluster analysis combined with assignment tests identified five clusters within the five communities.

**Conclusions:**

Some houses are colonized by insects from several genetic clusters after spraying, whereas other households are colonized predominately by insects from a single cluster. Significant population structure, measured by both *R*_ST_ and *F*_ST_, supports the hypothesis of poor dispersal ability and/or reduced migration of *T. infestans*. The high degree of genetic structure at small geographic scales, inferences from cluster analysis and assignment tests, and demographic data suggest reinfesting vectors are coming from nearby and from recrudescence (hatching of eggs that were laid before insecticide spraying). Suggestions for using these results in vector control strategies are made.

## Author Summary

Chagas disease is a protozoan infection caused by the parasite *Trypanosoma cruzi*. Chagas is prevalent throughout Central and South America, and it remains a chief concern in Bolivia. A movement that began in 1991 called the Southern Cone Initiative has been successful in reducing the incidence of Chagas disease in the Southern Cone countries of Argentina, Brazil, Chile, and Uruguay; but due to socio-economic and other factors, incidence remains high in Bolivia. The most important mode of transmission of *T. cruzi* to humans and other mammals is through feces of triatomine bugs. Thus, disease control and transmission prevention focus on elimination of triatomine vectors, and more specifically in Bolivia, it focuses on the elimination of *Triatoma infestans*. This study focuses on *T. infestans* in the Department of Chuquisaca, Bolivia. Ten highly variable microsatellite markers were used to analyze the population structure of insects collected in different towns. Statistical analyses show that *T. infestans* are highly structured, which means that they colonize on a small geographic scale. The results also suggest little active dispersal. These findings should be implemented during control efforts so that insecticide spraying focuses on geographic areas of colonization and re-colonization.

## Introduction

Chagas disease is a parasitic disease in which the pathogenic agent, *Trypanosoma cruzi* is transmitted by hematophagous insects of the sub-family Triatominae. *Triatoma infestans* is the major vector in the Andean highlands where the disease is endemic and has infected humans for over 9000 years [1]. Chagas disease is the most important parasitic disease in the Americas in terms of mortality and economic impact [2]. In Bolivia the endemic area covers 55% of the country and, in 1985, more than one million people were infected [3]. In 1991 a public health program, the Southern Cone Initiative was launched by the World Health Organization to eliminate vector populations [4], through spraying of houses and surrounding areas with pyrethroid insecticides [5]. In Argentina, Brazil, Chile, and Uruguay, *T. infestans* is exclusively domestic or peri-domestic, thus eradication of the vector in these regions, followed by vigilance against re-infestation, has proven largely successful in reducing transmission of *T. cruzi* and thus the prevalence of Chagas disease [6]. In contrast, in Bolivia the vectors occur in domestic, peri-domestic, and sylvatic environments [7]; thus, control of *T. infestans* in towns and homesteads is confounded by the possible re-infestation from surrounding sylvatic areas.

Molecular genetic research can help vector control programs by identifying and characterizing genetically distinct vector populations and then developing effective intervention strategies [8]. Several genetic markers including isozymes and the mitochondrial cytochrome b gene have proved useful in studying the genetic diversity of *T. infestans* [9],[10]; however, markers with more resolution would aid vector control efforts. DNA based microsatellite markers have been widely used in population studies because of their large polymorphism information content, widespread distribution in the eukaryotic genome and robust methodology.

To reduce transmission of Chagas disease, estimates of population differentiation are crucial to understand vector dispersal, sources of reinfestation, and gene flow; this genetic information is an important tool for effective management of insect control programs. Here we aimed to investigate the population genetic structure and inferred the source of colonization of vectors in the department of Chuquisaca, Bolivia using ten highly polymorphic microsatellite markers. The geographic region has high levels of human infection and house infestation and is located in a region thought to be the evolutionary origin of *T. infestans.*

## Methods

### Study sites and Triatomine sampling

**Figure 1:**
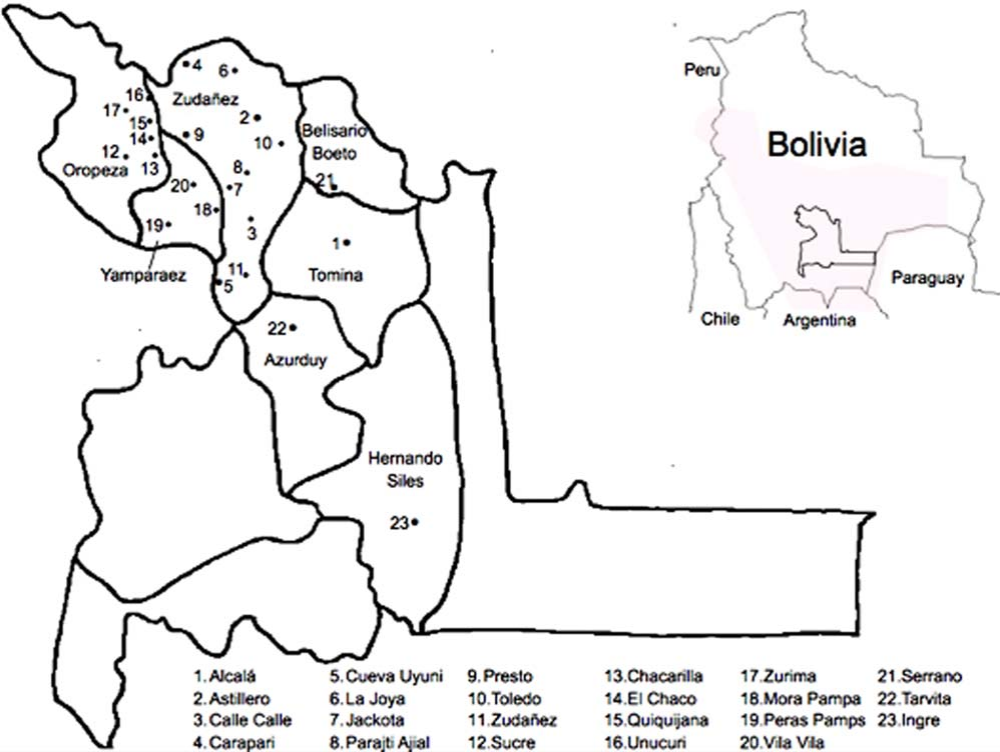
Distribution of *T. infestans* in Bolivia (shaded area) and locations of sample sites in Chuquisaca.

Insects were collected from 23 localities including both peri-urban (inhabited areas in the immediate vicinity of a city) and rural sites (less than 2000 inhabitants) in the provinces of Oropeza, Zudañez, Azurduy, Yamparaez, Tomina, Belisario Boeto and Hernando Siles within the Department of Chuquisaca, in the Bolivian highlands ranging from 1079 to 3020 meters above the sea level (Table 1, Figure 1). This area presents a broken topography with numerous valleys and small plateaus characterized by very diverse climates. In the Andean highlands, wheat is grown predominantly in small-scale, subsistence farming systems. In higher precipitation areas, potato is the preferred crop. Rainfall in these areas ranges from approximately 300 to 600 mm per year. In the Andean Plateau the average temperature is less than 10°C and there is less than 500 mm of annual precipitation. The Andean valleys present moderate climates, with average temperatures of 18°C and approximately 500 and 600 mm of rain every year. The relative humidity varies throughout the year, showing a similar pattern to the other climatic parameters. The majority of the vegetation in the plateau is grassy plain with a rich variety of grasses and dichotomous herbs, but also shrubs and some trees. The valleys contain fertile soils where vegetables, cereals and fruits are grown.

**Table 1:** Locations of the 23 populations of *Triatoma infestans* from Chuquisaca, Bolivia and their geographical and ecological grouping.

Province	n	Locality	N	Alt.	Latitude	Longitude	a	b	c	d	e
Tomina	1	Alcalá	2	2138	19°22′S	64°25′W	a1				
	2	Astillero	2	1922	19°30′S	64°11′W	a1				
	3	Calle Calle	1	2600	19°06′S	64°40′W	a1				
	4	Caraparí	1	1819	18°42′S	64°30′W	a1				
Zudanez	5	Cueva Uyuni	4	2449	19°25′S	64°54′W	a1				
	6	La Joya	3	2524	18°39′S	64°45′W	a1				
	7	Jackota	44	2425	19°04′S	64°48′W	a1	b		d	
	8	Parajti Ajial	3	2433	19°03′S	64°45′W	a1				
	9	Presto	3	2513	18°55′S	64°56′W	a1				
	10	Toledo	2	3009	19°10′S	65°05′W	a1				
	11	Zudáñez	2	2619	19°07′S	64°42′W	a1				
Oropeza	12	Sucre	37	2783	19°02′S	65°15′W	a2	b			
	13	Chacarilla	2	2544	18°53′S	65°05′W	a2				
	14	El Chaco	9	2381	18°52′S	65°05′W	a2	b			
	15	Quiqui-jana	1	2847	18°49′S	65°03′W	a2				
	16	Uñucurí	1	2648	18°46′S	64°57′W	a2				
	17	Zurima	78	2514	18°45′S	65°05′W	a2	b	c		e
Yamparaez	18	Mora Pampa	2	2403	19°43′S	64°22′W	a2				
	19	Peras Pampa	2	2419	18°56′S	65°06′W	a2				
	20	Vila Vila	1	3020	19°06′S	64°52′W	a2				
B. Boeto	21	Serrano	25	2235	19°06′S	64°22′W	a1	b			
Azurduy	22	Tarvita	3	2722	19°49′S	64°31′W	a1				
H. Siles	23	Ingre	2	1079	20°36′S	63°56′W	a1				

Alt. = meters above sea level, a) Eastern (a1, low altitude) vs. Western (a2, high altitude), b) 5 communities <100 Km apart, c) 7 houses in Zurima, d) corral in Jackota, e) domestic vs. peridomestic habitats, n = locality identification, N = number of insects.

Specimens of *T. infestans* included in the present study were a mixture of nymphs and adults, collected from inside as well as the immediate vicinity of homes. Collections were made in the months of the Southern hemisphere summer 2002, spring 2005 and fall 2005. Forty-four insects came from a single corral in the community of Jackota in the province of Zudañez, 78 insects were collected in the community of Zurima in the province of Oropeza, and 37 were collected in Sucre the capital and main city of Chuquisaca located in the province of Oropeza. The remaining 71 insects came from collections in 20 localities throughout Chuquisaca. All insects included in the study were identified as *T. infestans* using taxonomic keys [11]. Insects from the first collection were frozen live. Those from subsequent collections were placed in 95% ethanol while alive. Specimens then were sent to Vermont, USA for molecular analysis.

### Molecular analysis

DNA was extracted from three legs or 25 mg of tissue obtained from the posterior part of the abdomen of a given specimen using the Qiagen DNeasy DNA extraction kit (Qiagen, Inc., Valencia, CA). Care was taken to avoid sampling from the mid-abdomen as the stomach may inhibit the PCR reaction [12].

#### Microsatellite and genotyping system

We used ten previously published microsatellite markers: TiA02, TiC02, TiC08, TiC09, TiD09, TiE02, TiE12, TiF03, TiF11 and TiG03 [13]. To allow us to amplify and analyze all 10 loci in a single multiplex reaction, primers for three loci (TiC08* ‘5-AAG CAA ATA TTT TGT GTG TGT GTG -3”, TiD09* ‘5 –GGT CTT GGA TTT TAT GGG TTA TTT T -3’, and TiF03* ‘5 –CAC ACA AAC ACT TAA ACA CAC ACA A -3’) were modified so that the PCR product size did not overlap with other products of the same size range and fluorescence label. Our PCR reactions used the Qiagen Multiplex PCR kit (Qiagen, Inc., Valencia, CA). Template DNA (50–100 ng), primers and molecular biology grade water were added to the 2× multiplex PCR master mix to a final volume of 25 µL. The concentration of each primer was adjusted to permit good readings of the fluorescent peaks, modified by the addition of a fluorescence label and produced PCR products with the number of base pairs as follows: TiA02, 0.138, HEX, 173–225; TiC02, 0.138, HEX, 157–211; TiC08*, 0.276, 6-FAM, 110–144; TiC09, 0.552, NED, 125–159; TiD09*, 0.276, NED, 294–342; TiE02, 0.138, HEX, 147–167; TiE12, 0.276, HEX, 303–321; TiF03*, 0.276, 6-FAM, 215–269; TiF11, 0.138, NED, 256–280; and TiG03, 0.552, HEX, 200–250. The amplification protocol consisted of an initial step of 15 min at 95°C to activate the DNA polymerase and denature the template DNA, followed by 30 cycles of 30 sec at 94°C, 90 sec at 55°C, 60 sec at 72°C, and a final extension step of 10 min at 72°C. All reactions were carried out in a Techne TC-512 thermocycler (Techne Duxford, Cambridge, MA). PCR products were diluted 1/10 in distilled water then analyzed on an ABI Prism 3100 genetic analyzer using a ROX labeled size standard. Genotypes were read using GeneMapper^TM^ version 4.0 software (Applied Biosystems, Foster City, CA). The multiple PCR products were analyzed on an ABI Prism 3100 genetic analyzer using a ROX labeled size standard. Genotypes were read using GeneMapper^TM^ version 4.0 (Applied Biosystems, Foster City, CA).

### Data analysis

We investigated population genetic structure at both ecological and geographic levels (Table 1 a–e). Ecological grouping included: Eastern, low altitude (97 individuals) vs. Western, high altitude (133 individuals) regions (Table 1 a) and domestic (36 individuals) vs. peri-domestic habitats (42 individuals) within Zurima (Table 1 e). The geographic groupings included: among 5 communities within a 100 Km diameter with a total of 193 individuals (Table 1 b), among 7 households within a 750 m diameter (defined as a house and the associated peri-domestic buildings and corrals, with 4, 7, 14, 7, 6, 11 and 3 insects respectively) within Zurima (Table 1 c), and 36 nymphs from a single corral in Jackota (Table 1 d). Four insects from a household in Zurima were collected in 2002 before spraying, all other specimens were sampled in 2005, up to 6 months after spraying and were re-infesting insects.

#### Estimating population structure

Genetic population structure was investigated using hierarchical analysis of molecular variance (AMOVA) [14] for the model structures shown in Table 1 (a–e), using both F_ST_ (based on the infinite alleles model, IAM) and R_ST_ (based on a stepwise mutation model, SMM) [15] using the software Arlequin version 3.1 [16]. Values for the two statistics were tested for significant departure from zero using permutation tests contained within the software package. Nei's genetic distances among the 5 communities in group b (Table 1) were calculated and a UPGMA dendogram was constructed.

#### Isolation by distance

To test for isolation by distance we performed a regression analysis of Slatkin's [17] linearized *F*_ST_, (*F*_ST_/1−*F*_ST_) onto the natural log of geographic distance. In addition, a Mantel test [18] was used to assess the correlation between geographic distances among localities and differences in altitude with respect to Nei's unbiased standard genetic distances with 10000 random permutations using Arlequin version 3.1. The analysis was done using the five localities with sample size >8 (mean = 38.60, Table 1 b).

Pairwise multilocus estimates of the effective number of migrants (*Nm*) based on private alleles [17], were estimated using the software Genepop 3.4 [19], because this technique is more conservative than estimates based on *R*_ST_ for the sample sizes used in our study. The results were adjusted for diploid data, *M = 2Nm*. Based on the assignment test (see below) results from the software Structure [20] which showed mismatches between source and assigned populations from as far apart as 100 Km, we conducted a second Mantel test with 1000 permutations to determine the relationship between geographical distances and number of migrants (*Nm*).

#### Assignment test

We performed a Bayesian clustering analysis using the software Structure [20]. The number of populations, K was determined using the admixture ancestry model and correlated allele frequencies, testing K = 2 to 6 for the five communities in group b (Table 1) and K = 2 to 8 for the 7 households in Zurima. Each replicate was run 100,000 times following a burn-in of 50,000 runs. Individuals were assigned a cluster if the proportion ancestry ≥0.7; when no cluster was ≥0.7, the individual was unassigned.

#### Relatedness

The average relatedness (*r*) among groups of insects within households from Zurima (12 households), Serrano (4) and Ingre (1) was determined using the software Relatedness version 5.0 [21]. All individuals from a single household were used to define Px and Py.

## Results

### Population genetic structure

There was significant genetic differentiation among populations based on *R*_ST_ and *F*_ST_ estimates for all hierarchical levels analyzed (Table 2). Between low altitude East and high altitude West, R_ST_ and F_ST_ are statistically significant (R_ST = _0.08, *F*_ST_ = 0.02); both measures are also significant among the five communities <100 Km apart (R_ST_ = 0.12, *F*_ST_ = 0.06) and among houses in Zurima (R_ST_ = 0.14, *F*_ST_ = 0.07). We also observed significant differentiation between domestic and peri-domestic populations within the community of Zurima (R_ST_ = 0.05, *F*_ST_ = 0.03).

**Table 2:** Results of analysis of molecular variance (AMOVA) at ten microsatellite loci.

Model	Variation among	d.f.	R_ST_ *	P-value	F_ST_*	P-value
a) Between East and West	populations within regions	1	7.74 (R_ST_:0.08)	<0.001	2.40 (F_ST_:0.02)	<0.001
	individuals within populations	226	34.94 (R_IS_:0.38)	<0.001	37.07 (F_IS_:0.38)	<0.001
	Within individuals	230	57.31 (R_IT_:0.43)	<0.001	60.52 (F_IT_:0.40)	<0.001
b) Between localities <100 Km apart	populations	4	12.35 (R_ST_:0.12)	<0.001	6.55 (F_ST_: 0.06)	<0.001
	individuals within populations	188	30.99 (R_IS_:0.35)	<0.001	33.80 (F_IS_:0.36)	<0.001
	Within individuals	193	56.65 (R_IT_:0.43)	<0.001	59.65 (F_IT_:0.40)	<0.001
c) Houses 100–1500 m apart	houses	6	14.15 (R_ST_:0.14)	<0.001	6.69 (F_ST_:0.07)	0.02
	individuals within houses	48	12.62 (R_IS_:0.14)	0.006	31.13 (F_IS_:0.33)	<0.001
	individuals	52	73.23 (R_IT_:0.27)	<0.001	62.18 (F_IT_:0.38)	<0.001
d) single Corral	individuals	36	49.26 (R_IS_:0.49)	<0.001	35.83 (F_IS_:0.36)	<0.001
e) domestic vs peri-domestic	habitats	1	5.06 (R_ST_:0.05)	<0.001	2.69 (F_ST_:0.03)	<0.001
	individuals within habitats	76	32.00 (R_IS_:0.34)	<0.001	37.77 (F_IS_:0.39)	<0.001
	Within individuals	78	62.94 (R_IT_:0.37)	<0.001	59.54 (F_IT_:0.41)	<0.001

For population information see Table 1. Significance levels based on 1000 permutations, * = percent of variation.

**Figure 2:**
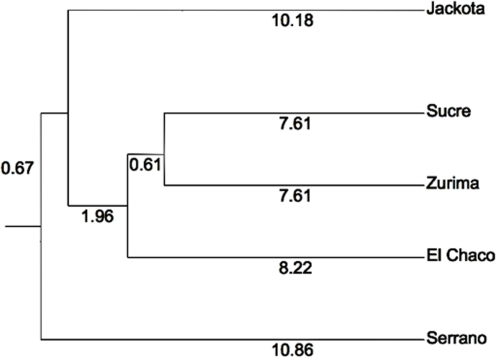
Dendogram of 5 populations of *T. infestans* from Chuquisaca, Bolivia. Dendogram based on Nei's (1978) standard genetic distance using the UPGMA method modified from the Neighbor procedure of Phylip version 3.5. Numbers are distances between nodes.

Although East and West were genetically differentiated, we did not observe a trend towards higher diversity at higher altitude when we compared the Western populations with a mean altitude of 2600 m, which comprises the provinces of Oropeza and Yamparaez, with the Eastern populations having a mean altitude of 2300 m which includes the provinces of Zudañez, Belisario Boeto, Azurduy, Tomina and Hernando Siles. The mean number of alleles per locus was 15.3±2.23 and 13.6±2.31 at the high and low altitudes respectively (t-test, P>0.05). The dendogram based on Nei's genetic distances showed a cluster comprising populations from Zurima, El Chaco and Sucre differentiated from a sister cluster with the Jackota population (Figure 2). These two clusters were well differentiated from a cluster containing populations from the more distant Serrano (Table 3). Pairwise estimates of *R*_ST_ and *F*_ST_ among communities (Table 4) support the conclusion that El Chaco, Zurima and Sucre are genetically similar to each other and that these communities differ from Jackota and Serrano. Within the town of Zurima, the estimates of *R*_ST_ and *F*_ST_ among the 7 households are shown in Table 5. With respect to *R*_ST_, households 4 and 5 are the most different from other households. These households represent peri-domestic samples and their difference from the other households is also shown by the significant difference among habitats (Table 2 e).

**Table 3:** Assignment of individuals from 5 localities to genetic populations through Bayesian analysis.

Population	N	Cluster 1	Cluster 2	Cluster 3	Cluster 4	Cluster 5	NA	%A
Jackota	44	1	2	2	32	1	6	86
Sucre	37	25	1	1	1	3	6	84
El Chaco	9	5	1	0	0	1	2	78
Zurima	78	10	24	24	0	5	15	81
Serrano	25	1	1	1	0	18	4	84
Total	193	42	29	28	33	28	33	

The numbered clusters represent distinct groups identified by Bayesian cluster analysis, using Structure [20]. Each cell contains the number of individuals from each population assigned to the cluster with q≥0.70.

NA = Not Assigned = number of individuals not assigned to any cluster.

%A = percent assigned to a genetic cluster.

### Assignment test

Five clusters were identified among the 5 communities (Table 3). When assigning individuals to genetic populations based on these communities, 78–86% of the individuals were assigned. The clusters represent insects with similar genotypes. Assignment tests can be viewed in terms of the number and evenness of communities in a single cluster and with respect to the number and evenness of clusters represented in a single community. Cluster 1 was a mixture of insects from the three close localities, Sucre, El Chaco and Zurima. The other four clusters contained insects from primarily one locality: clusters 2 and 3 were primarily from Zurima (24/29 = 83% and 24/28 = 86% respectively); cluster 4 from Jackota (32/33 = 97%) and cluster 5 from Serrano (18/28 = 64%). About 15–20% of the insects from each community were not assigned. From the community perspective, most of the insects from four of the communities are from a single genetic group: Jackota (73% from cluster 4), Sucre (67% from cluster 1), El Chaco (56% from cluster 1) and Serrano (72% from cluster 5). Zurima contains a mixture of groups, 13% group 1, 31% from group 2 and another 31% from group 3.

At the household level, five genetic clusters were identified from the seven households (Table 6). Insects from households 1, 2, 5 and half of those from household 7 were collected in peri-domestic settings, all the others came from domestic structures. The assignment test was quite successful for some households (100% assigned), yet for other households none of the insects were assigned. There does not seem to be any tendency for insects collected from domestic vs. peri-domestic sites to be assigned. With respect to the life stage and household of origin for the insects in each cluster, clusters 3 and 5 were mostly from a single household (86% and 100% respectively) with cluster 5 being composed only of the most geographically isolated insects and cluster 3 containing 5 nymphs and one adult from household 3 along with one adult male from household 6. Cluster 2 contains insects from 5 of the 7 households and cluster 1 contains insects coming from 4 households. Cluster 4 contains only nymphs, five from household 3 and four from household 2. The fifth cluster was a mix of adults and nymphs coming exclusively from Z-6. All four insects from the pre-spraying collection were not assigned to any cluster (Z-1) (Table 6).

### Relatedness

Relatedness of insects in nine out of seventeen houses was not significantly different from 0 (Table 7). From these nine households, in six cases at least one adult was collected and in three cases only nymphs were collected. For one household (S-1), *r*<0 (P<0.05) indicating significant outcrossing. For seven houses *r*>0 (P<0.05). A value of *r*≈0.25 (half sibs) was obtained for four households, and although the relatedness was similar in these households, the composition of the insect collection varied (1 site only adults 1 site only nymphs and 2 sites a mix of adults and nymphs). For the sites with the highest relatedness values (***r***≈0.33, 0.44 and 0.48), in 2 houses a single adult and 2–4 nymphs were collected and for one household only nymphs were collected.

### Number of migrants

The estimates of the effective number of migrants per generation, *Nm*, among towns <40 Km apart was higher (2.03) compared with those among more distant communities (1.42) and among houses within the town of Zurima (0.99). The Mantel test of isolation by distance revealed a non-significant correlation between Slatkin's linearized *F*_ST_ and *Nm* vs. the natural log of geographic distance (*R* ^2^ = 0.001, *P* = 0.294; *R* ^2^ = −0.184, *P* = 0.725 respectively). Non-significant results were also observed when applying the Mantel test for a correlation between Nei's genetic distances and geographic distances among populations (*R* ^2^ = 0.00056, *P* = 0.135), and altitude (*R* ^2^ = −0.000012, *P* = 0.548). The Mantel tests had low power because of the small samples within many of the communities.

## Discussion

Our study region is an ecologically diverse but geographically small valley–mountain environment in the department of Chuquisaca in Southern Bolivia. This region has high levels of house infestation and vector and human *T. cruzi* infection [22]–[24]. The use of microsatellite loci, now routine in many insect population genetic studies because they are inherently more polymorphic than allozyme loci and generally not targets of selection, allows us to detect population structure with more statistical power [25].

### Structure among *T. infestans* populations

Previous studies on population genetics and morphometry of *T. infestans* from Bolivia have found geographical variation in patterns of population structure in this vector; therefore we examined distinct ecological and geographic hierarchical groups ranging from a single goat corral to comparing western and eastern regions of Chuquisaca.

Genetic analysis over twenty-three localities throughout the department of Chuquisaca have revealed moderate but highly significant levels of genetic variation among populations. Both F_ST_ and R_ST_ showed differentiation even within a community. Previous study in the same area using a mitochondrial *cyt b* gene [10] failed to verify significant genetic diversity comparing distant rural and peri-urban settings. However, significant differentiation was revealed when populations from Chuquisaca (Andean) were compared with non-Andean populations from Brazil, Argentina and the Bolivian Chaco. Cytogenetic [26] and allozyme [9] studies have also confirmed genetic differences between *T. infestans* from highlands (>1800 m) and lowlands (<500 m). We examined insects from eastern and western Chuquisaca that significantly differ in altitude, both groups are >2000 m, and we detected significant differentiation at this ecological level.

In our study, R_ST_ values were larger than F_ST_, suggesting polymorphism is high and rates of migration are low [27]. The IAM-based estimates (F_ST_) indicate lower differentiation because they do not distinguish among shared alleles in different populations that are not identical by descent. Similar values of R_ST_ and F_ST_ are only to be expected when mutation rates are negligible in comparison to migration and drift. When the SMM contributes to population differentiation, R_ST_ values should be larger than F_ST_ values [28]. When comparing the 5 communities (Table 4), in general, pairwise R_ST_>F_ST_ suggesting that mutation contributes to differences at this geographic level. However, there is no such pattern for pairwaise R_ST_ and F_ST_ among households suggesting that mutation does not contributes much to differentiation at this level.

**Table 4:** Pairwise estimates of multilocus R_ST_ (below diagonal) and F_ST_ (above diagonal) between samples of *T. infestans* from Chuquisaca.

	Jackota	Sucre	El Chaco	Zurima	Serrano
Jackota		0.10***	0.09***	0.09***	0.09***
Sucre	0.14***		0.05*	0.02***	0.05***
El Chaco	0.22***	0.02		0.04**	0.07***
Zurima	0.22***	0.01	0.04		0.03***
Serrano	0.10***	0.05***	0.19***	0.10***	

Results of permutation test of R_ST_ or F_ST_>0: *P<0.05, **P<0.01, ***P<0.001.

### Population structure and the panmictic unit

As suggested by R_ST_>F_ST_, *T. infestans* has a low capacity for active dispersal [29] but can passively disperse over long distances when associated with human migration. It seems that this has been the structuring pattern of *T. infestans* in Chuquisaca. In our study, the results of the assignment of individuals to genetic clusters (Table 3) shows the assignment of insects to genetic populations located >100 Km apart.

Several studies using isozymes have examined population structure in *T. infestans* and report variation among regions in the spatial scale of population differentiation. Variation in population structure among regions was established using twelve isozymes [9],[30]. There was significant differentiation of *T. infestans* populations between villages located 50 Km apart in Vallegrande, Santa Cruz yet in the Yungas of La Paz, populations only a few Km apart showed significant differences. Using 19 isozyme loci, significant differences in allele frequencies between populations separated by 20 Km were found in central Bolivia [31], but this study failed to detect differentiation between sylvatic and domestic populations of *T. infestans*. By contrast, incipient differentiation between sylvatic and domestic populations was revealed using morphometry of the head capsule [32]. Other studies [33] have indicated that the panmictic unit may be no larger than a single household, based on the finding of significant differentiation within households in Yungas, Bolivia. Differences have also been detected between geographically close populations based on wing geometric morphometry [34].

The results of our study show significant population structure among communities. These results are supported by cluster analysis, which identified the geographically isolated communities as separate clusters (Jackota and Serrano, Table 3); however the closer communities are not as genetically distinct (Sucre, El Chaco and Zurima, Table 3). If migration depends on habitat quality, when insects find favorable conditions at the microhabitat level it can reduce their dispersal tendency and consequently reduce gene flow. Within the community of Zurima we sampled 7 houses and statistical analysis estimated 5 clusters within an area of 750 m diameter. These results suggest the single household is not the panmictic unit in this area of Chuquisaca and is in accordance with a study on dispersal capacity in the towns of Trinidad and Mercedes, Argentina, that clustered the source of re-infestation at ∼500 meters [35].

The isolation-by-distance tests based on allozyme markers in populations from several areas in Bolivia and Peru found a positive correlation between genetic and geographic distances [9]. We found no evidence of isolation by distance within this area of Chuquisaca. Differences between the two studies may result because our study had low statistical power due to sampling a relatively small number of communities, few samples per community and microsatellite data, because of the high number of alleles, require large sample sizes. However, the non-significant results may also be because our study covers a small geographic area of Chuquisaca characterized by a high human migration rate in the last 40 years [36].

### Control implications

Previous studies [37] identified unique local characteristics in landscape and vegetation, distances between houses, the abundance of bugs and hosts, and presence of many peri-domiciliary structures in conjunction with the existence of sylvatic populations as contributing to spatial patterns of re-infestation. Identification of the source of re-colonizers can direct control programs in the surveillance phase. We have found significant differentiation at the household level in populations from Chuquisaca, Bolivia. Cluster analysis, relatedness estimates and life stage data can be combined to understand pre-spraying population dynamics and infer patterns of re-colonization.

Within Zurima, individuals collected in the most geographically isolated household (Z-6) were assigned to one cluster. The relatedness of insects in Z-6 was significantly greater than 0 (Z-6, *r*>0.17, c.i. = 0.15, Table 7). Eight of the nine adults and the two nymphs in Z-6 were assigned to a single cluster, but this house also had insects from two other clusters.

**Table 5:** Pairwise estimates of multilocus R_ST_ (below diagonal) and F_ST_ (above diagonal) for *T. infestans* from 7 households in Zurima.

	Z-1	Z-2	Z-3	Z-4	Z-5	Z-6	Z-7
Z-1		0.07	0.10**	0.13***	0.11*	0.14**	0.07
Z-2	0.03		0.08***	0.11***	0.09*	0.13***	0.05
Z-3	0.12	0.11*		0.05*	0.05*	0.09***	0.04
Z-4	0.25***	0.25***	0.07*		0.01	0.14***	0.10
Z-5	0.11	0.19**	0.16***	0.07*		0.08**	0.09
Z-6	0.03*	0.03	0.20***	0.29***	0.14*		0.10*
Z-7	0.003*	0.10	0.12	0.31***	0.33*	0.14	

Results of permutation test of R_ST_ or F_ST_>0: *P<0.05, **P<0.01, ***P<0.001.

**Table 6:** Assignment of individuals from 7 households in Zurima to genetic populations through Bayesian analysis.

Household	Habitat	N	Cluster 1	Cluster 2	Cluster 3	Cluster 4	Cluster 5	NA	%A
Z-1	P	4	0	0	0	0	0	4	0
Z-2	P	7	0	1	0	4	0	2	71
Z-3	D	14	1	1	6	5	0	1	93
Z-4	D	7	1	2	0	0	0	4	43
Z-5	P	6	1	1	0	0	0	4	33
Z-6	D	11	0	1	1	0	8	1	91
Z-7	P/D	3	3	0	0	0	0	0	100
Total		52	6	6	7	9	8	16	

The numbered clusters represent distinct groups identified by Bayesian cluster analysis, using Structure [20]. Each cell contains the number of individuals from each population assigned to the cluster with q≥0.70.

H = habitat, D, domestic; P, peri-domestic; N = total number of individuals from each household, NA = Not Assigned = number of individuals not assigned to any cluster.

%A = percent assigned to a genetic cluster.

**Table 7:** Average relatedness and confidence intervals among *T. infestans* collected from households from 3 communities in Chuquisaca, Bolivia.

Household	Habitat	N	A?	AF	AM	N1	N2	N3	N4	N5	R	C.I.
S-1	D	2	1						1		−0.19	0.14
S-4	D	4	4								0.03	0.07
Z-12	D	3	2						1		0.05	0.08
Z-3	D	14		1		1	5	4	2	1	0.05	0.07
Z-2	P	7		2	5						0.10	0.13
Z-7	P/D	3		1				1		1	0.14	0.30
Z-9	D	6			1	2				3	0.16	0.21
Z-6	D	11		3	6					2	0.17	0.15
Z-4	D	7					4	3			0.19	0.21
Z-1	P	4	1				2		1		0.23	0.37
Z-5	P	6		3	3						0.23	0.21
S-3	D	6	1					1	2	2	0.26	0.21
I-1	D	2							1	1	0.26	0.36
S-2	D	5				1	2			2	0.27	0.13
Z-11	D	5			1	1	1	2			0.33	0.31
Z-8	D	3	1					1	1		0.44	0.42
Z-10	D	3					2		1		0.48	0.45

Households are ordered from highest to lowest relatedness.

*: S = Serrano, Z = Zurima, I = Ingre, D = domestic, P = peri-domestic, N = total number of individuals from each household, A? = adult, AM = adult male, AF = adult female, N1-N5 = first to fifth instars R = relatedness (<0 = outbred, 0 = random, 0.125 first cousins, 0.25 half sibs, 0.5 = full sibs, parent-offspring, C.I. = 95% confidence interval for relatedness estimate.

The reinfestation patterns for individual houses are quite variable including repeated colonization from several sources (Z-2, seven peri-domestic adults, *r*≈0.10, c.i. 0.13, Table 7), a single multiply mated female (S-3, 1 adult 5 nymphs, *r*≈0.26, c.i. = 0.21, Table 7), multiple colonization from a single source (Z-5, 3 males and 3 females, *r*≈0.23, c.i. = 0.21, Table 7), recrudescence of full sibs (Z-10, 3 nymphs, *r*≈0.48, c.i. = 0.45, Table 7) and recrudescence of unrelated eggs (Z-3, 14 insects mostly nymphs, *r*≈0.05, c.i. = 0.07, Table 7). Of course there are multiple possibilities for each household and these inferences are to show the range of possibilities, not to infer a given scenario for a specific household.

The presence of adults in many households less than 6 months after spraying suggests that for many cases, structures around human habitations may be playing a key role as the source of insects invading houses. The presence of nymphs in houses where no adults were found suggests recrudescence. Hence, recrudescence from a residual population and colonists from peri-domicile structures, rather than reinvasion from surrounding localities, seems to be a probable explanation of the source of re-colonists found during surveillance activities in this area.

The variety of results suggest that continuous surveillance consisting of analyzing relatedness among reinfesting insects at the household level is critical to maintain insect free houses and optimize insecticide spraying in this region.

## Supporting Information

Alternative Language Abstract S1 Translation of the abstract into Spanish by Juan Carlos Pizarro.
(0.05 MB PDF)
