# Supplementary material for: Microsatellites Reveal a High Population Structure in Triatoma infestans from Chuquisaca, Bolivia
Source: PLoS Negl Trop Dis. 2008 Mar 26;2(3):e202. doi: 10.1371/journal.pntd.0000202 (PMC2268005; doi:10.1371/journal.pntd.0000202)
Supplement: Alternative Language Abstract S1 — Translation of the abstract into Spanish by Juan Carlos Pizarro. (0.05 MB PDF) [file pntd.0000202.s001.pdf]

Translation of the abstract into Spanish by Juan Carlos Pizarro

Resumen

La estructura genética de poblaciones del *Triatoma infestans* (Hemiptera: Reduviidae), el principal vector de la enfermedad de Chagas en Bolivia fue investigada usando una estrategia jerárquica de muestreo. Usando 10 loci microsatelitales, se analizó un total de 230 insectos entre adultos y ninfas provenientes de 23 localidades a lo largo del Departamento de Chuquisaca, al sur de Bolivia. La estructura poblacional estimada usando el análisis de varianza molecular (AMOVA) para estimar los valores de F_{ST} (modelo de alelos infinitos) y de R_{ST} (modelo de mutaciones por pasos), fue significativa entre los insectos de las regiones del oeste y del este de Chuquisaca y entre los insectos capturados en hábitats domésticos y peridomésticos. La diferenciación genética en tres niveles geográficos jerárquicos fue significativa, incluso en el caso de viviendas adyacentes de una misma localidad (R_{ST} = 0.14, F_{ST} = 0.07). En una escala geográfica mayor, entre 5 comunidades localizadas a una distancia máxima de 100 km., el R_{ST} = 0.12 y el F_{ST} = 0.06. El análisis de conglomerados combinado con pruebas de asignación genética identificó 5 conglomerados en las 5 comunidades. Las pruebas de asignación sugieren que algunas casas son colonizadas por insectos provenientes de varios conglomerados genéticos, mientras que otras viviendas son colonizadas predominantemente por insectos de un solo conglomerado. La estructura poblacional significativa medida tanto por los valores de R_{ST} como por los de F_{ST} apoyan la hipótesis de una pobre habilidad de dispersión del *T. infestans*. El alto grado de estructura genética en un espacio geográfico pequeño y las inferencias producto del análisis de

conglomerados y de las pruebas de asignación, combinadas con datos demográficos, sugieren que los vectores que reinfestan las viviendas proceden de los alrededores así como de la recrudescencia (eclosión de huevos puestos antes del fumigado con insecticida). Se realizan sugerencias para el uso de estos resultados en las estrategias de control vectorial.
